# A method for estimating the outer exposure of dairy cows to deoxynivalenol (DON) and zearalenone (ZEN) as a precondition for risk assessment based on inner exposure with toxin residue levels in blood and urine as indicators

**DOI:** 10.1007/s12550-024-00533-6

**Published:** 2024-04-26

**Authors:** Sven Dänicke, Janine Saltzmann, Benno Waurich, Adriana Wöckel, Wolf Wippermann, Guntram Hermenau, Julia Wittich, Erik Bannert, Fanny Rachidi, Peter Hufe, Detlef May, Hermann Swalve, Alexander Starke, Melanie Schären-Bannert

**Affiliations:** 1 Institute of Animal Nutrition, Friedrich-Loeffler-Institut (FLI), Federal Research Institute for Animal Health, 38116 Braunschweig, Germany; 2 Institute of Agricultural and Nutritional Sciences, University of Halle, Halle, Germany; 3 Clinic for Ruminants and Swine, Faculty of Veterinary Medicine, Leipzig University, Leipzig, Germany; 4 LVAT—Institute for Animal Breeding and Husbandry, Groß Kreutz, Germany

**Keywords:** Deoxynivalenol, Zearalenone, Exposure, Mycotoxin

## Abstract

DON and ZEN residues in the blood and urine of dairy cows can be used to predict the outer exposure to DON and ZEN expressed per kilogram diet for a risk evaluation based on comparisons to critical dietary concentrations. This method was used to evaluate the exposure of dairy cows from 12 farms located in Brandenburg, Germany, fed rations with unknown DON and ZEN concentrations (*N* = 244). The corresponding diet concentrations predicted by different methods from analyzed blood and urine samples varied significantly amongst farms from 0 to 1.6 mg/kg for DON and 0 to 3.0 mg/kg for ZEN at a reference dry matter content of 88% but independently of lactational state (*post-partum* vs. early lactation). This significant variation was noticed below the critical dietary DON concentration of 5 mg/kg, while the ZEN concentration in one farm exceeded the critical ZEN level of 0.5 mg/kg markedly. Predicted DON concentrations of rations increased with the proportion of maize silage, while the high ZEN concentration found in one farm was most likely related to a higher proportion of sugar beet pulp supposedly highly contaminated by ZEN. Exceeding the critical dietary ZEN concentration and significant variations in DON contents below the critical level was not related to performance, reproductive performance, and health-related traits of cows. For a more consistent evaluation of possible associations between the inner exposure of cows to DON and ZEN, more frequent longitudinal observations of both mycotoxin residue levels and performance and health traits are required.

## Introduction

Dairy cows are exposed to mycotoxins via the daily feed ration generally composed of roughage and concentrate feed. Feeding practices mainly include mixing these principal components to a total mixed ration (TMR) or to a partial mixed ration (PMR) to be completed by additional milking performance-dependent concentrate feed amounts.

The outer exposure to important mycotoxins such as deoxynivalenol (DON) and zearalenone (ZEN) is determined by the concentration of these toxins in feed and by the intake of dry matter (DMI) relative to body weight (BW). However, under practical feeding conditions where cows are kept in groups, the individual DMI, and usually also BW, remains unknown. Moreover, mycotoxin distribution across feed components and batches and mixing homogeneity of concentrate feed and final TMR/PMR are significant sources of uncertainty for the evaluation of the external exposure of cows. On the other hand, guidance values for critical diet concentrations of 5 mg DON and 0.5 mg ZEN at a reference DM content of 88% have been established (European Commission 2006). As their application in feeding practice solely relies on representatively sampled and analyzed feed, the actual external exposure is largely variable and might not reflect the individual inner exposure. Indicators for the latter are mycotoxin residue levels in systemic blood circulation, but also in urine and milk as routes of mycotoxin elimination from the systemic circulation. Besides the fact that mycotoxin residue levels in physiological specimens are not only dependent on outer exposure but also on toxicokinetics, a diagnosis of individual inner exposure based on analyzing mycotoxin residues in blood and other physiological specimens might also be subject to variation. Despite this variability noticeable for external and internal exposure, there exist linear relationships between both enabling a prediction of individual external mycotoxin exposure and mycotoxin concentration in feed based on mycotoxin residue levels analyzed in physiological specimens (Dänicke et al. 2023). In the absence of established critical mycotoxin levels in physiological specimens, the predicted mycotoxin diet concentrations can be evaluated by comparing them with the mentioned guidance values for feed.

Against this backdrop, the aim of the present study was to assess the inner exposure of dairy cows to DON and ZEN based on toxin residue levels in blood and urine collected from 12 farms located in Brandenburg, Germany, and to predict the outer exposure including the corresponding diet concentrations as a precondition for risk assessment.

As DMI follows typical dynamics in the course of lactation (National Research Council 2001) and because of a supposedly higher vulnerability of *post-partum* cows to DON and ZEN exposure, sampled cows of each farm were subdivided into *post-partum* (PP) and early lactation (EL) cows.

We hypothesized that the inner exposure of cows would differ between farms and lactational stages due to different feeding and DMI levels, respectively. Furthermore, we tested the hypothesis that mycotoxin residues in blood and urine would correspond to milking and reproductive performance as well as to health traits as indicators for the toxic effects of DON and ZEN.

## Material and methods

### Description of sampling and data acquisition

A total of 244 Holstein cows were sampled for urine and blood from 12 farms in Brandenburg, Germany. The farm had an average size of 583 German Holstein cows (236–1383), an average milk production of 10561 kg per cow and year, and a 29.6% culling rate (excl. sales for breeding purposes). All farms implemented a total-confinement free-stall and total mixed ration (TMR) feeding system.

The cows were subdivided into *post-partum* (PP) and early lactation (EL) cows. This sampling schema mirrored a complete twelve-by-two two-factorial design. A total of 10 replications per group were targeted and achieved in 21 out of the 24 sub-groups. In two sub-groups were one and two samples less available, respectively, while from one farm, 20 samples were presented. Across all farms, groups PP and EL were 3.5 (0.9–13.9) and 11.9 (2.0–28.3) weeks in lactation (means, span in brackets), respectively, and in parities 3.0 (1.0–8.0) and 3.5 (1.0–9.0).

For the purpose of the present study, leftovers from blood and urine samples routinely collected during farm visits in the fourth quarter of 2018 and the first quarter of 2019 were used for mycotoxin residue analysis. Due to the retrospective character of the study, feed samples were not available for mycotoxin analysis. Information on feed composition corresponding to the blood and urine samples was provided by the farms.

Prior to sample collection, the cows were examined and the following traits were recorded: rectal temperature, body condition score (BCS, 1–5) (Edmonson et al. 1989), lameness score (1–5) (Rachidi et al. 2021), hygiene score (0–2) (adapted from Reneau et al. 2005), rumen filling, and stratification (0–3) (Dirksen et al. 1990). Urine mid-stream samples were collected by manual stimulation. Blood samples were collected from the coccygeal vein or artery using 10-mL serum and heparin tubes. Milk production was recorded, and milk samples were collected during the monthly official milk control. Samples were analyzed for fat %, protein %, and somatic cell count.

### Mycotoxin residue analysis in blood and urine

ZEN, DON, and their metabolites (indicated in Table 1) were simultaneously analyzed in blood plasma and urine by LC–MS/MS. The used analytical method is described in detail by Brezina et al. (2014).

**Table 1:** Mean rate of recoveries (%), LOD and LOQ (ng/mL) of ZEN, DON and their metabolites in measuring solution of bovine blood plasma and urine

	Blood plasma			Urine		
	Recovery ± SD	LOD	LOQ	Recovery ± SD	LOD	LOQ
ZEN	113 ± 11	0.01	0.03	97 ± 16	0.05	0.10
Alpha-ZEL	100 ± 8	0.15	0.51	99 ± 5	0.25	0.90
Beta-ZEL	104 ± 7	0.17	0.57	101 ± 11	0.60	1.95
ZAN	106 ± 2	0.07	0.22	103 ± 2	0.15	0.50
Alpha-ZAL	108 ± 4	0.03	0.12	93 ± 5	0.10	0.35
Beta-ZAL	127 ± 6	0.03	0.11	117 ± 19	0.15	0.45
DON	105 ± 8	0.19	0.65	108 ± 17	0.25	0.80
de-DON	100 ± 12	0.09	0.31	114 ± 19	0.25	0.85

*LOD* limit of detection, *LOQ* limit of quantification, *SD* standard deviation, *ZEN* zearalenone, *alpha-ZEL* alpha-zearalenol, *beta-ZEL* beta-zearalenol, *ZAN* zearalanone, *alpha-ZAL* alpha-zearalanol, *beta-ZAL* beta-zearalanol, *DON* deoxynivalenol, *de-DON *de-epoxy-DON

Before enzymatic incubation overnight with β-glucuronidase (type H-2 from *Helix pomatia*, Sigma-Aldrich, Steinheim, Germany), urine samples were diluted in a ratio of 1:5 with water. After the incubation step, which was implemented to record the sum of conjugated and unconjugated analytes, plasma and diluted urine samples were purified by solid phase extraction cartridges (Oasis HLB; Waters, Milford, MA, USA) (Winkler et al. 2014b, 2015b).

The specified LODs, LOQs, and recoveries of each analyte differentiated by the matrix are presented in Table 1. The obtained results were not corrected for recoveries, but results of urine samples were multiplied with the dilution factor of urine samples before the evaluation of the data.

### Calculations and statistics

Dietary DON and ZEN concentrations (always expressed at a DM content of 88%) were predicted by using two different types of regression equations. The first type linearly regresses the DON and ZEN residues detected in blood and urine (ng/mL) on the DON and ZEN exposure (µg/kg BW/d) (Eqs. 1–4), respectively, while the second type of equations uses the DON and ZEN concentrations (mg/kg diet at 88% DM) directly as response variables (Eqs. 5–8). Prediction equations were either already published by Dänicke et al. (2023) (Eqs. 1, 2, 5) or newly developed (Eqs. 3, 4, 6, 7, 8) for the present study using the databases described in this publication.

1 $$DON\;exposure=2.52\cdot DON\;residues\;in\;blood$$

$$[{r}^{2}=0.784, RSE=30.9\;\mu g/kg\;BW/d]$$

2 $$DON\;exposure=0.022\cdot DON\;residues\;in\;urine$$

$$[{r}^{2}=0.637, RSE=59.2\;\mu g/kg\;BW/d]$$

3 $$ZEN\;exposure=80.7\cdot ZEN\;residues\;in\;blood$$

$$[{r}^{2}=0.611, RSE=7.4\;\mu g/kg\;BW/d]$$

4 $$ZEN\;exposure=0.584\cdot ZEN\;residues\;in\;urine$$

$$[{r}^{2}=0.618, RSE=7.7\;\mu g/kg\;BW/d]$$

5 $$DON\;diet=0.07\cdot DON\;residues\;in\;blood$$

$$[{r}^{2}=0.784, RSE=1.22 mg/kg\;diet]$$

6 $$DON\;diet=0.00062\cdot DON\;residues\;in\;urine$$

$$[{r}^{2}=0.633, RSE=1.6 mg/kg\;diet]$$

7 $$ZEN\;diet=2.33\cdot ZEN\;residues\;in\;blood$$

$$[{r}^{2}=0.644, RSE=0.2 mg/kg\;diet]$$

8 $$ZEN\;diet=0.016\cdot ZEN\;residues\;in\;urine$$

$$[{r}^{2}=0.646, RSE=0.2 mg/kg\;diet]$$

DON/ZEN exposures used to establish Eqs. 1–4 were based on experimentally determined DMI, BW, and DON/ZEN concentrations of diet, and calculated as follows:

9 $$\begin{aligned} & DON\;or\;ZEN\;exposure\\&\;=\frac{DMI\cdot DON\;or\;ZEN\;concentration\;of\;diet\;DM}{BW} \end{aligned}$$

Both in establishing the prediction Eqs. 1–8 and their usage for the present farm screening, the DON and ZEN residues as predictor variables were expressed as the sum of all analytically determinable forms of DON and ZEN according to Eqs. 10 and 11.

10 $$\begin{aligned} DON\;residues&=DON+de-DON\\&\quad+conjugates\;(glucuronidated\;and\;sulfated\;DON\;and\;de-DON)\end{aligned}$$

11 $$\begin{aligned} ZEN\;residue=&\;ZEN+alpha-ZEL+beta-ZEL\\&+ZAN+alpha-ZAL+beta-ZAL\\&+conjugates\;(glucuronidated\;and\;sulfated\;ZEN\;and\;metabolites)\end{aligned}$$

In order to predict the DON and ZEN concentrations of diets from Eqs. 1–4 where the response variable is the exposure, Eq. 9 needs to be solved accordingly:

12 $$\begin{aligned} & DON\;or\;ZEN\;concentration\;of\;diet\;DM\\&\;=\frac{BW\cdot DON\;or\;ZEN\;exposure}{DMI} \end{aligned}$$

Using Eq. 12 requires the knowledge of DMI and BW which, however, are unknown under practical conditions. Therefore, both parameters were estimated by using the relationships to other traits recorded in the present project. Thus, DMI was predicted according to the National Research Council (2001) for Holstein cows:

13 $$DMI=(0.372\cdot FCM+0.0968\cdot {BW}^{0.75})\cdot (1-{e}^{\left(-0.192\cdot \left(WOL+3.67\right)\right)})$$

where FCM is the 4% fat-corrected milk yield (kg/day), BW is the body weight (kg), and WOL denotes the week of lactation. The term $\left(1- {{\text{e}}}^{\left(-0.192\cdot \left({\text{WOL}}+3.67\right)\right)}\right)$ accounts for depressed DMI during early lactation. BW necessary for prediction of DMI was estimated based on the equations published by Gruber (2015) as follows:

14 $$BW=((721.81-11.807\cdot MOL+1.3447\cdot {MOL}^{2})\cdot 0.92)\cdot {CF}_{Parity}$$

where MOL is the month of lactation influencing the BW in a quadratic manner in the course of the lactation. This prediction equation represents the average course across different breeds and varying parities. Thus, the BW was corrected by 0.92 to account for the breed Holstein cow and a parity depending variable correction factor (CF_Parity_).

All statistics were performed in the environment of RStudio, R version 4.2.1 (R Core Team 2021). Graphs were prepared using the package ggplot2 (Hadley Wickham 2016).

Regression Eqs. 3, 4, 6, 7, and 8 are estimated using the *lm* function of the package *stats* (R Core Team 2021).

Data were generally evaluated by a complete linear two-by-twelve two-factorial model with farm (levels A to M), lactational stage (levels EP and PP), and their interactions as fixed factors using the *lm* function of the package *stats* (R Core Team 2021). Function *kruskal.test* from package *stats* which performs a Kruskal–Wallis rank sum test was called when the residuals of the linear two-by-twelve two-factorial model demonstrated significant departures from normal distribution as evaluated by the simulation-based method *DHARMa* (Hartig 2017) generating readily interpretable scaled quantile residuals for fitted linear models.

When the Kruskal–Wallis rank sum test proved significant treatment effects, the pairwise Wilcoxon rank sum test was used to identify significant group differences using the function *pairwise.wilcox.test* of the package *stats* whereby *p*-values were adjusted according to Bonferroni to account for multiple comparisons. The function *multcompLetters* implemented in the library *multcompView* was used to convert the *p*-values into a character-based display in which characters identify groups that are significantly different/not different (Graves et al. 2019). Results were presented as box plots indicating the medians, the 25th and 75th percentiles limiting the boxes, the ± 1.5 interquartile range (IQR) as whiskers, and the individual observations and completed by the compact letter display.

When residual distribution suggested normal distribution, the results of the linear model were maintained and multiple Tukey-adjusted pairwise comparisons were additionally performed when fixed factors were significant (*p* < 0.05) using the function *emmeans* of the package *emmeans* (Lenth 2021). The function *cld* of the package *multcomp* was used to convert the *p*-values into a compact letter display (Hothorn et al. 2008). These letters were displayed along with the estimated marginal means (EMMs), the individual observations, and the confidence intervals as whiskers.

For examining possible associations between blood or urine DON and ZEN residues and traits recorded at the farm level possibly indicative of toxic effects, Spearman correlation coefficients were estimated using the function *correlation*, method = “spearman,” of the package *correlation* (Makowski et al. 2020). For the graphical presentation of selected correlations, the function *ggpairs* of the package *GGally* (Schloerke et al. 2021) was used.

To consider that contaminated feed batches are fed over longer periods of time and that chronic exposure has supposedly more pronounced effects on available parameters indicative of mycotoxin effects, the evaluation period included the time point of the blood sample in the middle and the weeks before and after the blood sample. For example, mean values of milking performance before and after the blood sample were used for studying correlations.

The explained two methods for predicting the dietary DON or ZEN concentrations (Eqs. 1–8) were further statistically evaluated whereby the method based on exposure was assumed as the gold standard. Precision and accuracy were evaluated by the concordance correlation coefficient (CCC) using the *CCC* function of the package *DescTools* (Signorell Andri et mult. al. 2022). Furthermore, agreement between both methods was visually assessed by the Bland–Altman method plotting the difference of corresponding observations, i.e., DON or ZEN concentration predicted by the two methods, against their means (Bland and Altman 1986). The scatter of these observations was descriptively evaluated aided by horizontal guiding lines indicating the mean of the difference covered by the range limited by the ± 1.96·standard deviation of that differences (Bland and Altman 1986).

## Results

### Feedstuff composition of diets

Information on diet compositions was based on feedstuff groups’ roughages and concentrate feeds including cereal grains and byproducts (Table 2). Principal roughage components in all farms were maize silage (27.5–69.3%), wilted lucerne silage (10.6–36.8%), and cereal straw/grass hay/rape straw/lucerne hay (0.6–5.9%). The aggregated fraction “concentrate feed/wet maize grains/spent grains/pressed pulp” varied from 6.8 to 39.5% of the total ration.

**Table 2:** Ration composition on dry matter basis (in %) according to farm (A–M) and lactational group (*PP*, *post-partum*; *EL*, early lactation)

Farm_Group	Maize silage	Wilted lucerne silage	Cereal straw/grass hay/rape straw/lucerne hay	Concentrate feed/wet maize grains/spent grains/pressed pulp
A_EL	65.6	10.6	0.6	23.2
A_PP	64.1	11.3	1.3	23.4
B_EL	46.0	31.3	0.6	22.1
B_PP	47.6	30.9	0.7	20.8
C_EL	42.2	29.6	2.1	26.1
C_PP	40.5	31.0	2.4	26.1
D_EL	49.3	24.6	1.3	24.8
D_PP	49.3	26.3	1.5	22.9
E_EL	34.9	24.4	1.1	39.5
E_PP	33.6	26.8	2.2	37.4
F_EL	43.3	36.8	1.3	18.6
F_PP	43.3	36.8	1.3	18.6
G_EL	27.5	33.0	1.1	38.4
G_PP	29.1	33.9	1.2	35.8
H_EL	54.5	20.1	1.2	24.2
H_PP	50.4	23.9	5.3	20.4
I_EL	49.8	12.5	5.2	32.5
I_PP	51.6	11.7	5.9	30.8
K_EL	69.3	23.1	0.8	6.8
K_PP	69.3	23.1	0.8	6.8
L_EL	44.8	23.4	4.9	26.9
L_PP	51.8	18.0	4.5	25.6
M_EL	37.4	34.0	1.7	27.0
M_PP	33.3	33.3	2.0	31.4

### DON and ZEN residues

**Fig. 1:**
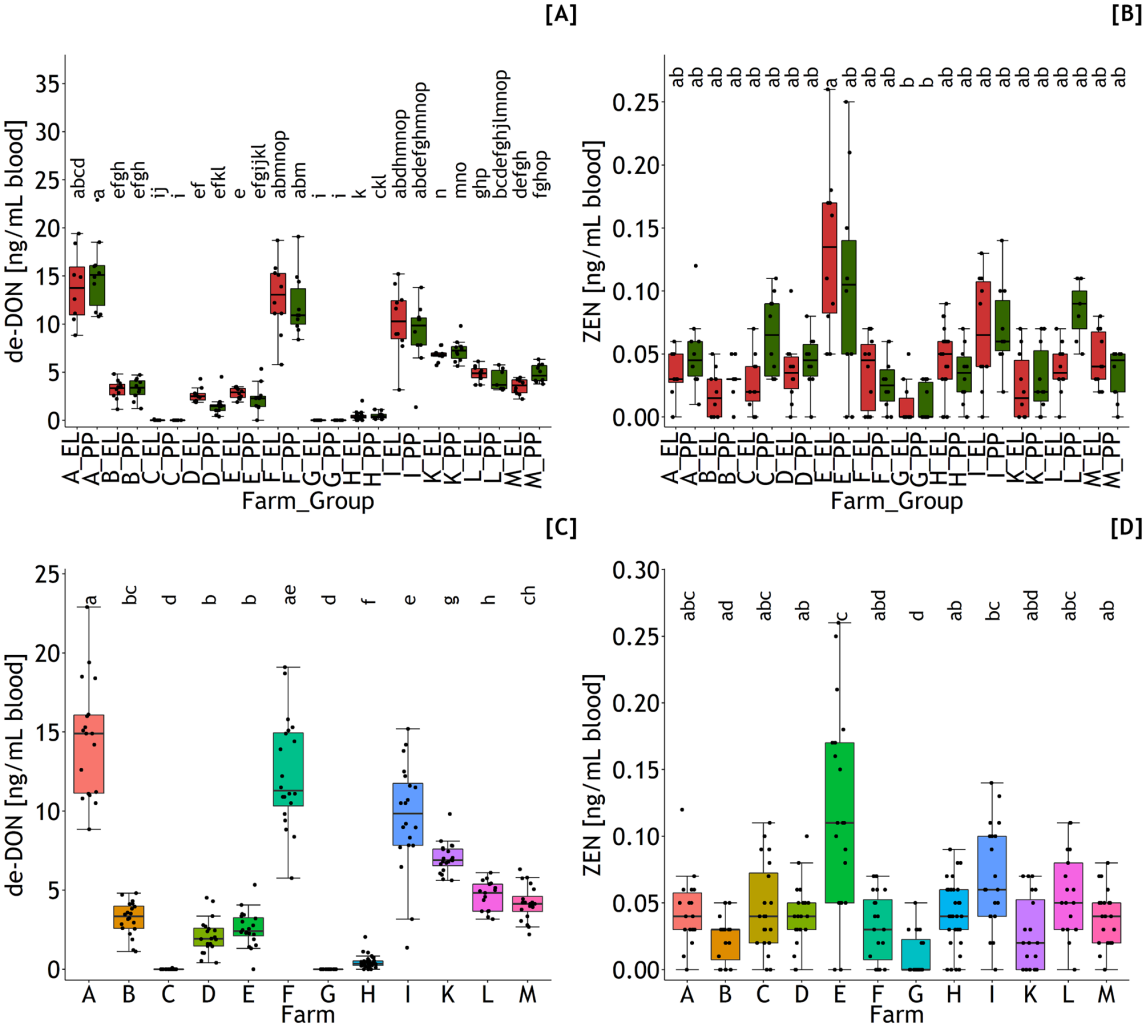
De-epoxy-deoxynivalenol (de-DON) and zearalenone (ZEN) concentrations in blood of dairy cows collected from 12 farms (A–M) separated for cows in early lactation (EL) and *post-partum* (PP) (**A** and **B**), and pooled over lactational state (**C** and **D**). Different letters indicate significant differences between farms and groups (*p* < 0.05)

In general, residue concentrations of ZEN and DON in blood and urine showed a non-normal distribution when the linear model was used for evaluating the data. Therefore, the nonparametric evaluation strategy was applied to these data, i.e., the Kruskal–Wallis rank sum test followed eventually by the pairwise Wilcoxon rank sum test. Moreover, none of the evaluated parameters proved significant differences between groups EE and PP within farms as exemplarily shown for de-DON and ZEN in blood (see Fig. 1A and B). Therefore, groups were pooled within farms whereby the number of multiple comparisons was reduced from 24 to 12.

### Blood

**Fig. 2:**
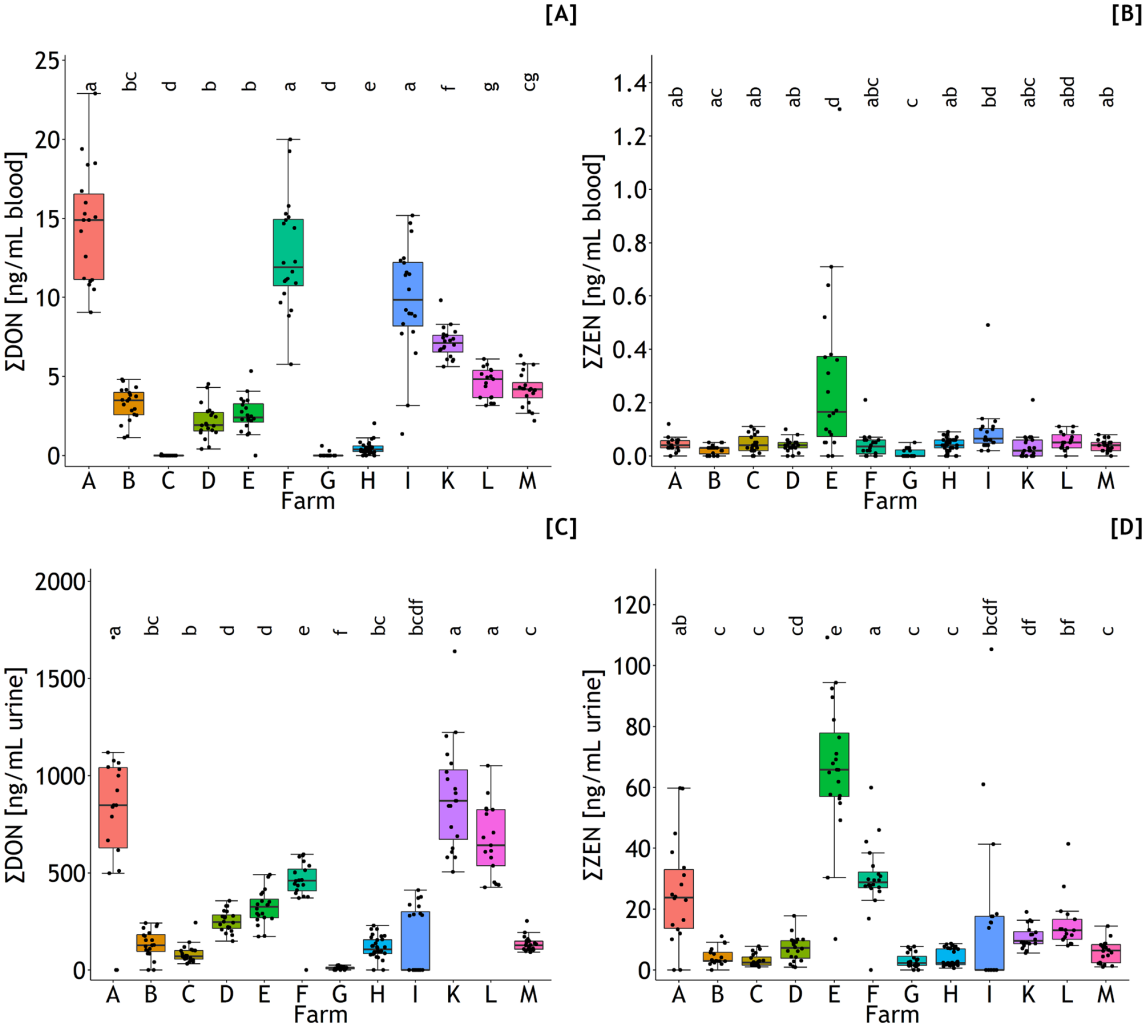
Sum of deoxynivalenol (DON) and zearalenone (ZEN) residue concentrations in blood (**A** and **B**) and urine (**C** and **D**) of dairy cows collected from 12 farms (A–M). Different letters indicate significant differences between farms (*p* < 0.05)

De-DON was the predominant DON residue in the blood (Fig. 1A and C) where 82.4% out of the 244 samples were proven to be positive. The maximum de-DON concentration of 22.9 ng/mL was found in farm A which differed significantly from all other farms except farm F (Fig. 1C). DON could be detected in only 12.2% of the samples, predominantly in the samples with higher de-DON levels. Summing DON and de-DON to the DON residues (Eq. 10) used for predicting the outer exposure to DON resulted in a similar farm ranking as outlined for de-DON (Fig. 2A).

From the analyzable ZEN metabolites, only ZEN (Fig. 1B and D), alpha-ZEL, and beta-ZEL with overall positive rates of 82.4, 1.6, and 4.1%, respectively, were detected. The maximum ZEN concentration of 0.26 ng/mL was found in farm E which was shown to differ significantly from ZEN levels found in farms B, D, F, G, H, K, and M (Fig. 1D). The higher ZEN concentrations found in farm E were associated with the presence of alpha- and beta-ZEL residues in a few samples nearly exclusively detected in this farm. ZEN residues (Eq. 11) were used as predictor variables for outer ZEN exposure and dietary ZEN concentration comprised of ZEN, alpha-ZEL, and beta-ZEL and demonstrated comparable farm ranking as shown for ZEN whereby the highest level found in farm E even clearer differed from the other farms (Fig. 2B).

### Urine

The concentrations of de-DON and DON in urine were generally higher compared to blood and reached maximum values of 1695 and 78 ng/mL, respectively (data not shown). Higher concentrations of DON residues in urine were associated with higher positive rates of 95.7% for de-DON and 53.7% for DON, respectively. The ranking of total DON residue levels in urine amongst farms (Fig. 2C) mirrored closely that in blood at a markedly higher level (Fig. 2A).

Beta-ZAL, a ZEN metabolite not detectable in blood, was found in 8.2% of the urine samples besides ZEN (data not shown), alpha-ZEL, and beta-ZEL with positive rates of 98.7, 36.8, and 60.6%, respectively. Beta-ZAL was detectable up to a maximum concentration of 4.6 ng/mL in 19 out of 20 urine samples collected from cows from farm E. While total ZEN residues in urine samples collected from farm E clearly underpinned the markedly high inner exposure of these cows to ZEN, the ranking amongst the other farms appeared to be strongly pronounced as compared to blood including significance relationships which probably resulted from the higher positive rates of ZEN metabolites in urine compared to blood.

### Predicted DON and ZEN concentrations of feed

Generally, the orders of magnitude of differences between farms for estimated DON and ZEN concentrations of diets are comparable to those reported for total DON and ZEN residues in blood and urine because of the linear factors (regression coefficients) linking residues with diet concentrations. The latter were estimated either indirectly through transforming the estimated exposures (Eqs. 1 to 4, “indirect” estimation) to the diet concentrations whereby the DMI and BW had additionally to be estimated (Eqs. 13 and 14) or directly through prediction equations linking residues directly to dietary DON and ZEN concentrations (Eqs. 5 to 8; “direct” estimation). In addition, corresponding DON and ZEN concentrations estimated indirectly or directly from plasma or urine residues were compared to evaluate the concordance between these two principal prediction methods by using CCC estimations and Bland–Altman plots.

**Fig. 3:**
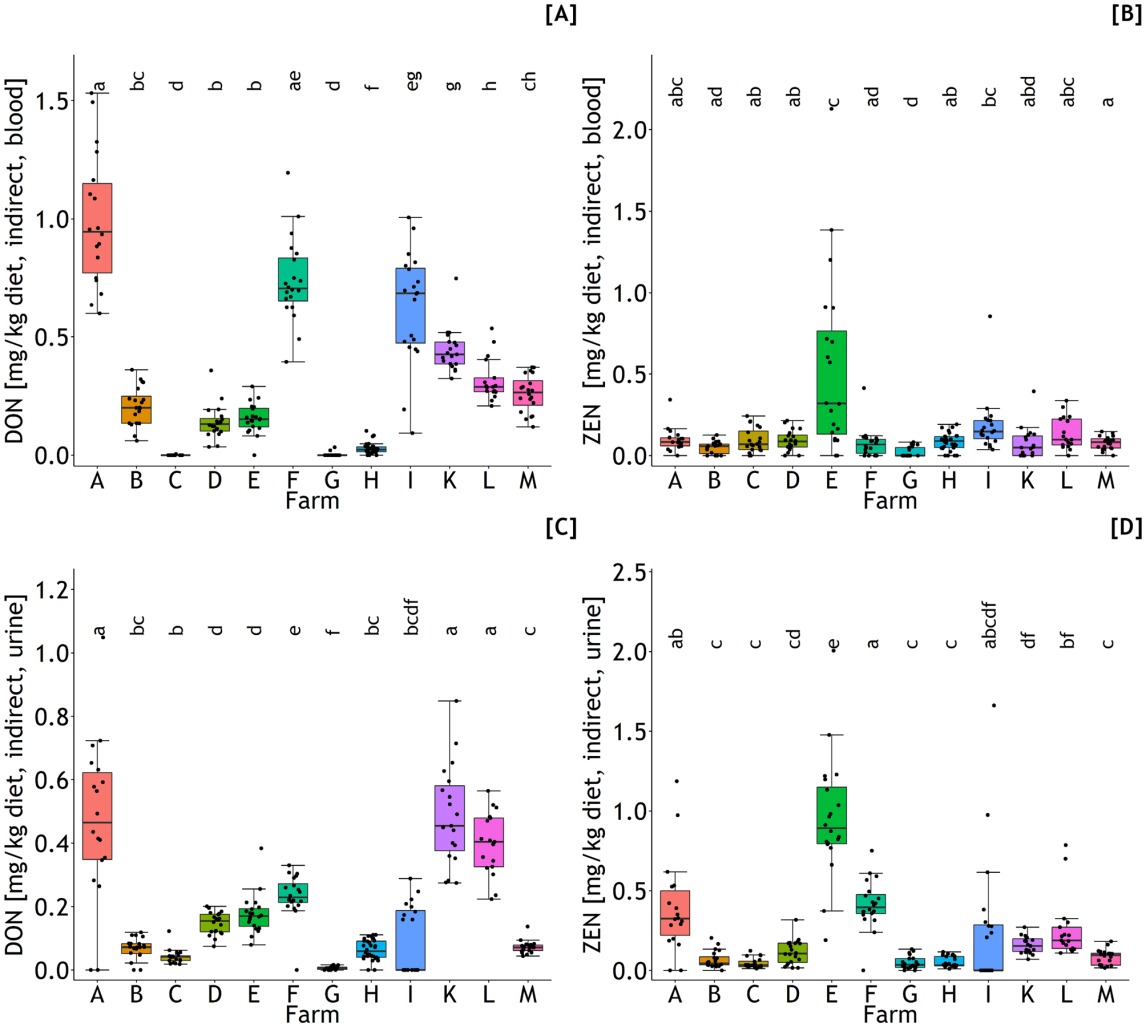
Deoxynivalenol (DON) and zearalenone (ZEN) concentrations of feed according to farm (A–M) estimated indirectly through exposures predicted from DON and ZEN residues in blood (**A** and **B**) or urine (**C** and **D**) whereby dry matter intakes (DMI) and body weights (BW) needed additionally to be estimated. Different letters indicate significant differences between farms (*p* < 0.05)

**Fig. 4:**
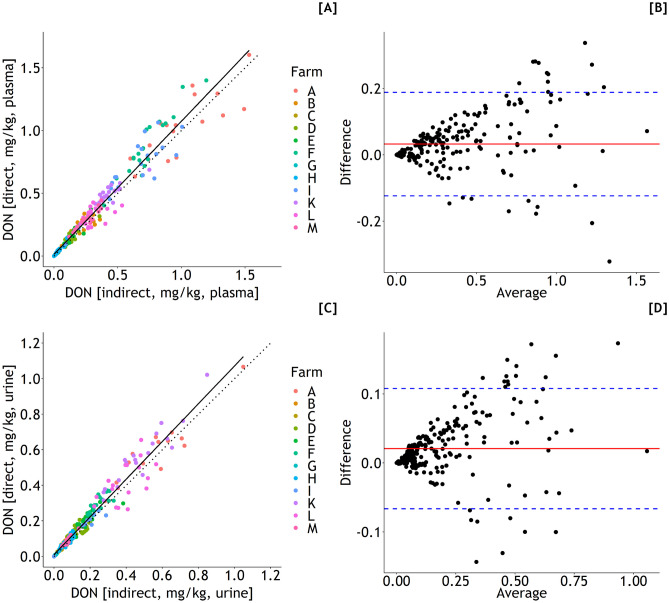
Associations between DON concentration of the diet based on DON exposure predicted by Eq. 1 (blood residues) (**A**) or 2 (urine residues) (**C**), and estimated dry matter intake (DMI) and body weight (BW) (indirect estimation of diet concentrations), and dietary DON concentration directly predicted by Eqs. 5 and 6, respectively. Observations are shown along with the regression lines (solid lines) and the 90° angle bisector (dotted lines) and as Bland–Altman plots (**B**, **D**), respectively. **A** *y* = 0.01 + 1.06*x*, *n* = 242, RSE = 0.08 mg/kg diet (solid line); Lin’s concordance correlation coefficient (CCC) = 0.968, Pearson’s correlation coefficient (*r*) = 0.992. **B** Mean difference of 0.03 (red solid line) ± 1.96·0.08 (standard deviation of difference, blue dashed lines) mg/kg diet. **C** *y* = 0.01 + 1.06*x*, *n* = 242, RSE = 0.04 mg/kg diet (solid line); Lin’s concordance correlation coefficient (CCC) = 0.968, Pearson’s correlation coefficient (*r*) = 0.991. **D** Mean difference of 0.02 (red solid line) ± 1.96·0.04 (standard deviation of difference, blue dashed lines) mg/kg diet

DON concentrations estimated indirectly and directly from plasma varied from 0 to 1.5 mg/kg diet (Eqs. 1 and 12, Fig. 3A) and 0 to 1.6 mg/kg diet (Eq. 5), respectively. The corresponding ranges for indirect and direct estimations of DON concentrations from urine were 0–1.0 mg/kg diet (Eqs. 2 and 12, Fig. 3A) and 0–1.1 mg/kg diet (Eq. 6). For both plasma and urine-based estimations, the concordance between indirect and direct predictions was reasonably good as indicated by Lin’s CCC of 0.968 and slopes of 1.06 for both cases (Fig. 4A and C) although the Bland–Altman plots suggested larger differences between indirect and direct predictions with increasing dietary DON concentrations (Fig. 4B and D).

**Fig. 5:**
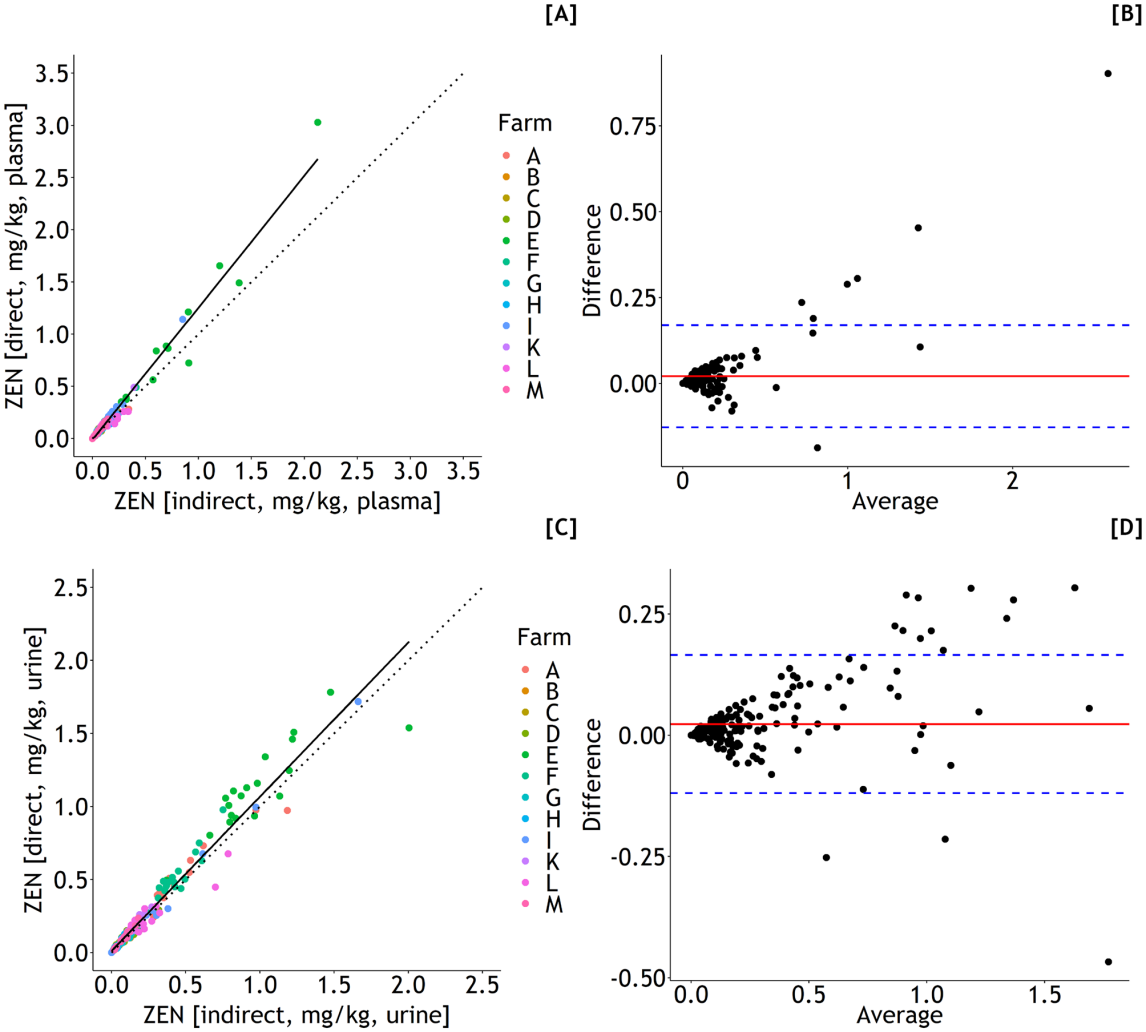
Associations between ZEN concentration of the diet based on DON exposure predicted by Eq. 3 (blood residues) (**A**) or 4 (urine residues) (**C**), and estimated dry matter intake (DMI) and body weight (BW) (indirect estimation of diet concentrations), and dietary ZEN concentration directly predicted by Eqs. 7 and 8, respectively. Observations are shown along with the regression lines (solid lines) and the 90° angle bisector (dotted lines) and as Bland–Altman plots (**B**, **D**), respectively. **A** *y* = 0.01 + 1.27*x*, *n* = 242, RSE = 0.05 mg/kg diet (solid line); Lin’s concordance correlation coefficient (CCC) = 0.95, Pearson’s correlation coefficient (*r*) = 0.956. **B** Mean difference of 0.02 (red solid line) ± 1.96·0.08 (standard deviation of difference, blue dashed lines) mg/kg diet. **C** *y* = 0.01 + 1.06*x*, *n* = 242, RSE = 0.04 mg/kg diet (solid line); Lin’s concordance correlation coefficient (CCC) = 0.973, Pearson’s correlation coefficient (*r*) = 0.994. **D** Mean difference of 0.02 (red solid line) ± 1.96·0.07 (standard deviation of difference, blue dashed lines) mg/kg diet

Indirectly and directly predicted ZEN concentrations in feed varied between 0 and 2.1 mg/kg (Eqs. 3 and 12, Fig. 3B) and 0 and 3.0 mg/kg (Eq. 7) for plasma and between 0 and 2.0 mg/kg (Eqs. 4 and 12, Fig. 3D) and 0 and 1.8 mg/kg (Eq. 8) for urine, respectively. Lin’s CCC amounted to 0.95 and 0.973 for comparisons of indirectly and directly predicted ZEN concentrations in feed based on plasma and urine residues, respectively, while the corresponding slopes were 1.27 and 1.06 suggesting larger differences between both prediction methods when plasma residues were used as predictor variables (Fig. 5A and C). Again, the Bland–Altman plots suggested larger deviations between both methods with increasing ZEN concentrations of diets (Fig. 5B and D).

### Production and health traits of cows

BCS was not significantly different amongst farms and groups with a mean value of 2.6 (1.5–4.0) (Table 3). Estimated BWs of PP and EL cows were comparable except for PP cows in farms F and L exhibiting a higher and lower BW than their EL counterparts, respectively, which explained the significant interactions between group and farm (Table 3).

**Table 3:** Cow and milk traits according to farm (A–M) and lactational group (*PP*, *post-partum*; *EL*, early lactation, *EL*). Values are given as means and standard errors (SE). Targeted number of replicates was *n* = 10

Farm_Group	Body weight (kg)		Body condition score		Rectal temperature (°C)		Hygiene score		Rumen fill score		Rumen stratification score		Non-return-rate		Lameness score		Milk protein (%)		Fat-to-protein ratio in milk		Somatic cell count of milk (× 1000/mL)	
	Mean	SE	Mean	SE	Mean	SE	Mean	SE	Mean	SE	Mean	SE	Mean	SE	Mean	SE	Mean	SE	Mean	SE	Mean	SE
A_EL	726	32	2.5	0.3	38.1	0.5	1.4	0.4	2.4	0.5	2.9	0.4	0.40	0.55	2.3	0.2	3.03	0.16	1.23	0.13	4.87	0.09
A_PP	683	22	2.5	0.5	38.8	0.4	1.2	0.5	1.9	0.5	2.2	0.6	0.60	0.55	2.2	0.1	3.03	0.20	1.42	0.26	4.96	0.12
B_EL	733	20	2.7	0.5	38.6	0.2	1.1	0.3	2.4	0.5	2.3	0.5	0.33	0.52	2.0	0.2	3.38	0.26	1.27	0.11	4.86	0.16
B_PP	677	20	2.7	0.5	38.9	0.3	1.4	0.4	1.7	0.4	2.0	0.5	0.43	0.53	1.8	0.1	3.43	0.38	1.28	0.21	4.77	0.18
C_EL	722	19	2.3	0.5	38.4	0.3	1.1	0.2	2.7	0.3	2.9	0.2	0.60	0.55	1.9	0.2	3.10	0.20	1.22	0.13	4.86	0.13
C_PP	682	22	2.4	0.7	38.6	0.5	1.6	0.2	1.9	0.7	2.2	0.6	0.25	0.50	2.1	0.2	3.32	0.25	1.44	0.15	4.76	0.13
D_EL	718	24	2.5	0.6	38.4	0.3	1.0	0.5	2.1	0.6	2.6	0.5	0.20	0.45	3.0	0.3	3.20	0.18	1.17	0.16	4.88	0.17
D_PP	671	15	2.8	0.7	38.8	0.5	0.8	0.3	2.2	0.5	2.5	0.6	0.50	0.55	2.9	0.3	3.25	0.23	1.28	0.11	4.95	0.15
E_EL	721	20	2.7	0.7	38.2	0.4	1.7	0.4	2.3	0.5	2.8	0.4	0.60	0.55	2.4	0.2	3.36	0.29	1.12	0.16	4.86	0.09
E_PP	686	16	2.6	0.6	38.3	0.6	1.5	0.4	2.4	0.5	2.7	0.5	0.75	0.50	2.8	0.3	3.34	0.32	1.24	0.10	4.77	0.17
F_EL	700	30	2.6	0.2	38.5	0.2	1.1	0.4	1.9	0.3	1.9	0.3	0.38	0.52	1.4	0.2	3.33	0.16	1.14	0.15	4.88	0.13
F_PP	699	17	2.9	0.4	38.5	0.1	0.9	0.3	1.9	0.2	2.0	0.2	0.57	0.53	2.1	0.2	3.42	0.23	1.14	0.15	4.83	0.17
G_EL	715	20	2.6	0.5	38.3	0.2	1.0	0.4	2.3	0.5	2.8	0.4	0.40	0.55	2.1	0.2	3.37	0.18	1.09	0.06	4.73	0.12
G_PP	664	15	3.0	0.6	38.4	0.4	0.7	0.3	2.4	0.5	2.9	0.3	0.86	0.38	1.4	0.2	3.35	0.25	1.25	0.17	4.86	0.10
H_EL	723	21	2.6	0.6	38.3	0.4	1.4	0.3	2.8	0.4	2.6	0.5	0.40	0.55	2.1	0.1	3.33	0.22	1.24	0.15	4.77	0.12
H_PP	686	17	2.5	0.6	38.9	0.2	1.2	0.3	2.3	0.5	2.4	0.5	0.50	0.71	2.2	0.2	3.24	0.22	1.30	0.11	4.78	0.15
I_EL	711	24	2.5	0.5	38.6	0.4	1.3	0.3	2.0	0.5	2.7	0.5	0.75	0.50	1.8	0.3	2.96	0.24	1.22	0.16	4.74	0.17
I_PP	686	23	2.7	0.8	38.7	0.3	1.1	0.4	1.9	0.3	2.7	0.5	1.00	0.00	1.7	0.3	3.17	0.33	1.51	0.45	4.79	0.16
K_EL	720	18	2.3	0.5	38.6	0.2	1.5	0.4	2.5	0.5	2.5	0.5	0.33	0.52	1.7	0.2	3.34	0.13	1.08	0.16	4.80	0.08
K_PP	677	18	2.9	0.5	38.6	0.4	1.3	0.5	2.6	0.5	2.3	0.5	0.33	0.52	1.7	0.2	3.30	0.33	1.25	0.16	4.87	0.13
L_EL	731	17	2.4	0.6	38.4	0.2	1.0	0.2	2.5	0.5	2.0	0.5	0.60	0.55	2.7	0.2	3.05	0.18	1.25	0.13	4.81	0.13
L_PP	642	40	2.3	0.6	38.7	0.4	1.7	0.5	2.4	0.9	2.3	0.5	0.60	0.55	2.3	0.3	3.24	0.23	1.38	0.20	4.72	0.33
M_EL	718	20	2.6	0.5	38.7	0.3	0.9	0.3	2.3	0.4	2.3	0.5	0.44	0.53	§		3.16	0.17	1.24	0.05	4.77	0.09
M_PP	690	17	2.4	0.4	38.4	0.4	0.8	0.3	2.1	0.6	2.1	0.3	0.43	0.53	3.0	0.4	3.19	0.26	1.23	0.12	4.84	0.14
*p*-values																						
Farm (F)	0.386		0.180		0.003		<0.001		<0.001		<0.001				<0.001		<0.001		<0.001		0.007	
Group (G)	0.012		0.101		<0.001		0.593		0.001		0.004				0.921		0.112		<0.001		0.799	
FxG	0.014		0.219		<0.001		<0.001		0.016		0.060				0.138		0.518		0.121		0.129	
KW													0.899									

^§^No data available; *KW* Kruskal–Wallis rank sum test

The rectal temperature averaged at 38.5 °C and varied inconsistently between 37.3 and 39.7 °C amongst farms and groups giving rise to significant interactions between farm and group (Table 3).

Hygiene scores differed between farms with variable rankings between groups within farms resulting in significant interactions between farm and group at an overall range of 0.5–2.0 (Table 3).

**Fig. 6:**
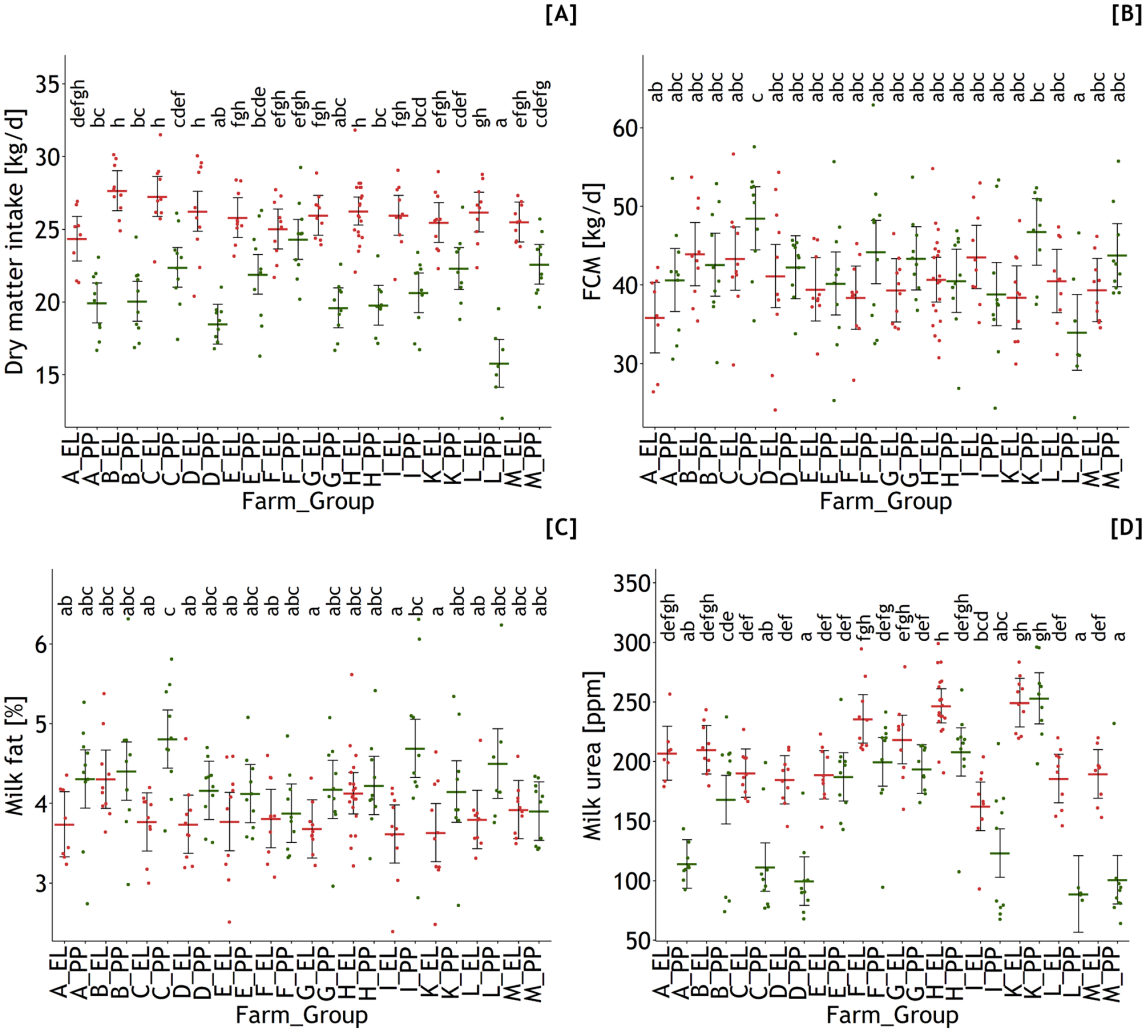
Estimated dry matter intake (DMI, **A**), measured fat-corrected milk yield (FCM, **B**), milk fat (**C**)**,** and milk urea content (**D**) evaluated for farm (**A–M**) and lactational group (EL, early lactation; PP, *post-partum*). Different letters indicate significant differences between groups and farms (*p* < 0.05)

Estimated DMI was lower in nearly all PP groups compared to their EL counterparts except farm F where cows of both groups reached a comparable level (*p*_farm_ < 0.001, *p*_group_ < 0.001, *p*_farmxgroup_ < 0.001) (Fig. 6).

PP cows of farms A, B, and H imposed through a rumen fill score approximately 0.5–0.7 points lower than their EL farm counterparts while only marginal differences between groups were detected in the other farms causing significant interactions between farm and group. The rumen stratification score mirrored farm and group differences as described for rumen fill score whereby the interactions occurred just as a trend (Table 3).

Lameness score differed significantly amongst farms but independently of group and reached a mean score of 3.0 (2–5) in farm D, compared to farm K where the lowest mean score of 1.7 (1–3) was observed (Table 3).

Milk yield corrected for a milk fat content of 4% varied inconsistently amongst farms and groups without a clear ranking between groups PP and EL (*p*_farm_ = 0.026, *p*_group_ = 0.030, *p*_farmxgroup_ = 0.014) (Fig. 6). Group PP from farm C reached the highest FCM of 48.5 kg/day with group PP from farm L with the lowest FCM of 34 kg/day.

Milk fat content was significantly influenced by group and farm in an interactive manner (*p*_farm_ = 0.097, *p*_group_ < 0.001, *p*_farmxgroup_ = 0.036) whereby most of the PP groups showed higher milk fat contents compared to their EL counterparts excepting those in farms B, F, H, and M displaying similar milk fat contents in both groups (Fig. 6).

Mean milk protein content of 3.2% varied significantly from 2.4 to 4.1% between farms irrespective of group assignment (Table 3). Based on the mostly higher milk fat contents in group PP and the group-independent milk protein content, the resulting milk fat-to-protein ratios closely mirrored the variations in milk fat content although the interactions failed to reach significance (Table 3). The mean fat-to-protein ratios amounted to 1.3 (0.8–2.6) and 1.2 (0.8–1.7) in groups PP and EL, respectively.

Milk urea content was lower in most of the PP groups but reached the level observed in EL groups in a few cases (*p*_farm_ < 0.001, *p*_group_ < 0.001, *p*_farmxgroup_ < 0.001) (Fig. 6).

Although there were significant farm differences in the somatic cell count (SCC) of milk, the variation was noticed at a comparable level between 4.8 and 4.9 × 1000 cells/mL (Table 3).

A total of 64 out of 130 cows across all farms and groups were not presented again for insemination 56 days after first insemination resulting in a non-return rate (NRR) of 49.2%. NRR was not significantly influenced by farm or group (Table 3). This reproductive trait was evaluated during the weeks after sampling blood and urine.

**Fig. 7:**
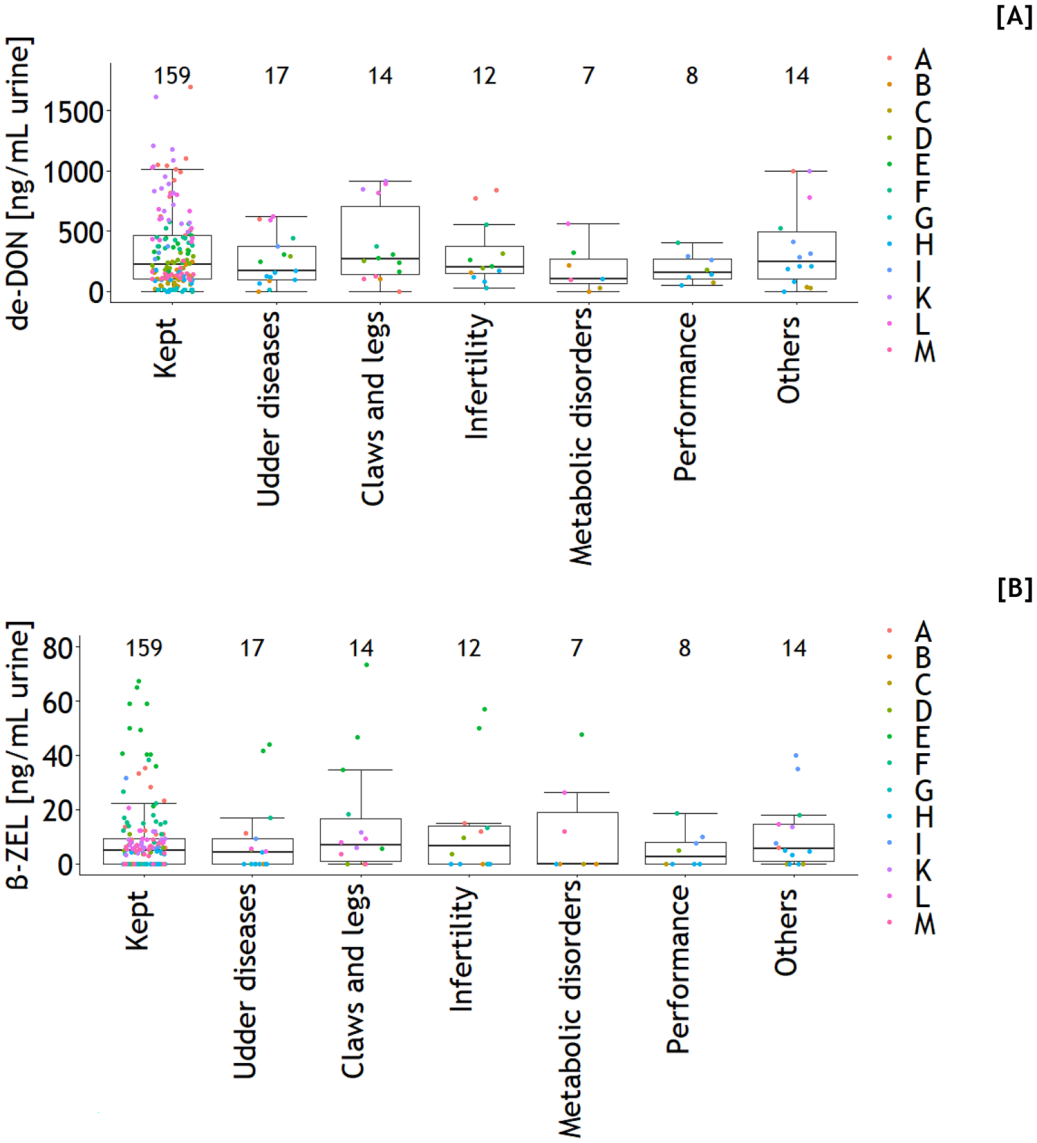
Assigning individual de-epoxy-deoxynivalenol (de-DON) and beta-zearalenol (beta-ZEL) concentrations in urine to culling reasons according to farms

The fate of the cows during the weeks after blood and urine sampling was classified as cows kept at farms (*n* = 159) and leaving the farms due to udder diseases (*n* = 17), claws and leg diseases (*n* = 14), infertility (*n* = 12), metabolic disorders (*n* = 7), low performance (*n* = 8), and other reasons (*n* = 14). An assignment of individual cows to de-DON and beta-ZEL concentrations in urine revealed no significant differences between these classes (*p* > 0.05) (Fig. 7).

### Correlations and regressions

**Fig. 8:**
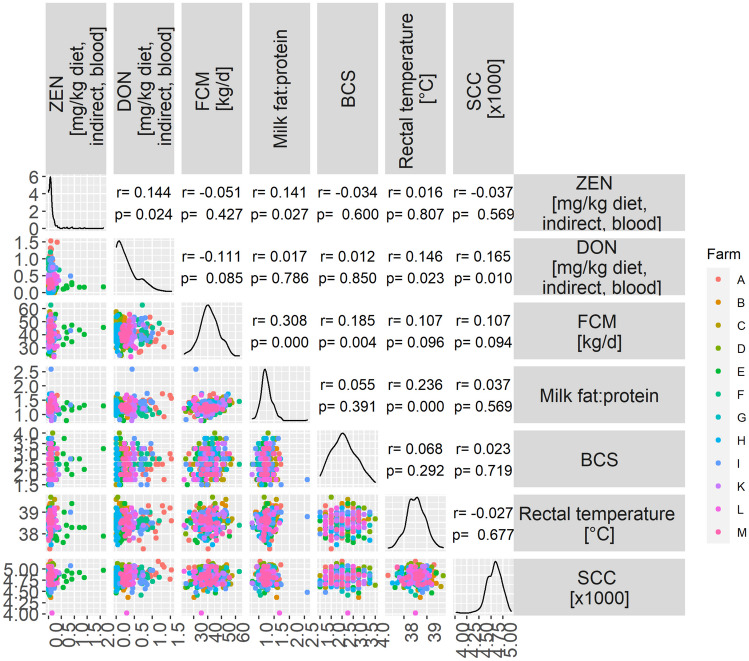
Spearman’s rank correlation coefficients, frequency distributions, and scatter plots of observations for selected traits. Abbreviations: DON, deoxynivalenol; ZEN, zearalenone; FCM, fat-corrected milk yield; BCS, body condition score; SCC, somatic cell count in milk

To identify possible associations between production or health traits and mycotoxin exposure indicators, Spearman's rank correlation coefficients were estimated (Fig. 8). Generally, all indicators of mycotoxin exposure, including mycotoxin residue levels in urine and blood, estimated DON and ZEN concentrations in the diets, etc. correlated quite well with each other. The same is true for some of the production and health traits. For example, both BCS and SCC were significantly positively correlated to FCM. However, the correlations between production/health traits and indicators for mycotoxin exposure were generally low and insignificant in most cases. For example, although estimated DON concentration in diet was significantly positively correlated with SCC and rectal temperature, the correlation coefficients were only 0.165 and 0.146, respectively. As a tendency, FCM and estimated DON concentration in diet were negatively correlated (*r* = − 0.111; *p* = 0.085).

**Fig. 9:**
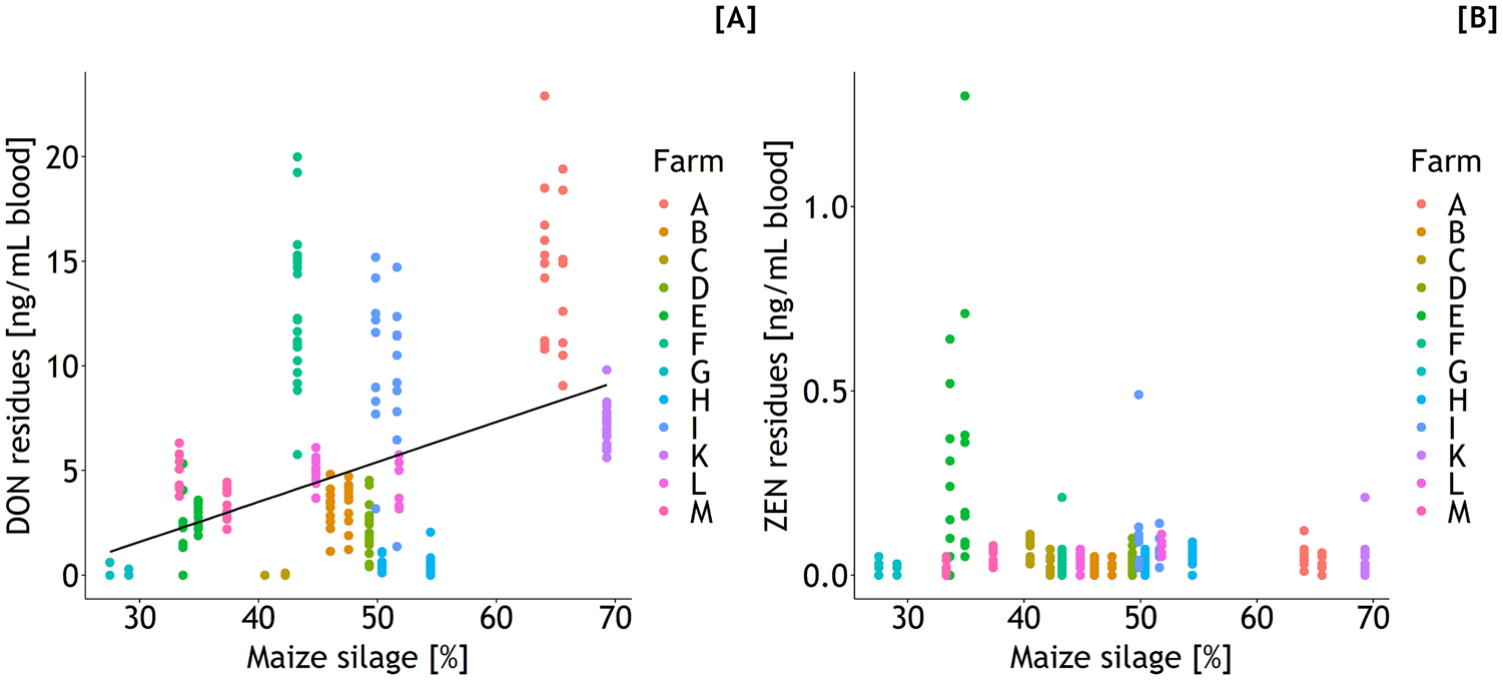
Deoxynivalenol (DON residues = − 4.14^p<0.05^ + 0.19 ^p<0.05^·*x*, *r*^2^ = 0.182 ^p<0.05^, residual standard error = 4.6 ng/mL; **A**) and zearalenone (ZEN, **B**) residue concentrations in blood in dependence on maize silage proportion of the daily ration

Feedstuff diet composition correlated moderately with mycotoxin residue levels in blood and urine. Of particular interest were the correlations between the proportions of maize silage which correlated moderately positively with DON residues in plasma (*r* = 0.427, *p* < 0.05) and urine (*r* = 0.631, *p* < 0.05) but only weakly and negatively with ZEN residues in plasma (*r* = − 0.165, *p* < 0.05) and urine (*r* = − 0.113, *p* > 0.05). On the other hand, inverse correlations between the proportions of the aggregated feed fraction “concentrate feed/wet maize grains/spent grains/pressed pulp” (individual proportions not specified) and DON residues in plasma (*r* = − 0.349, *p* < 0.05) and urine (*r* = − 0.481, *p* < 0.05) and ZEN residues in plasma (*r* = 0.295, *p* < 0.05) and urine (*r* = 0.307, *p* > 0.05) were found. Regressing the DON residues in blood on the maize silage proportion suggested an increase of 0.19 ng/mL for each increase in maize silage proportion of 1% (Fig. 9).

**Fig. 10:**
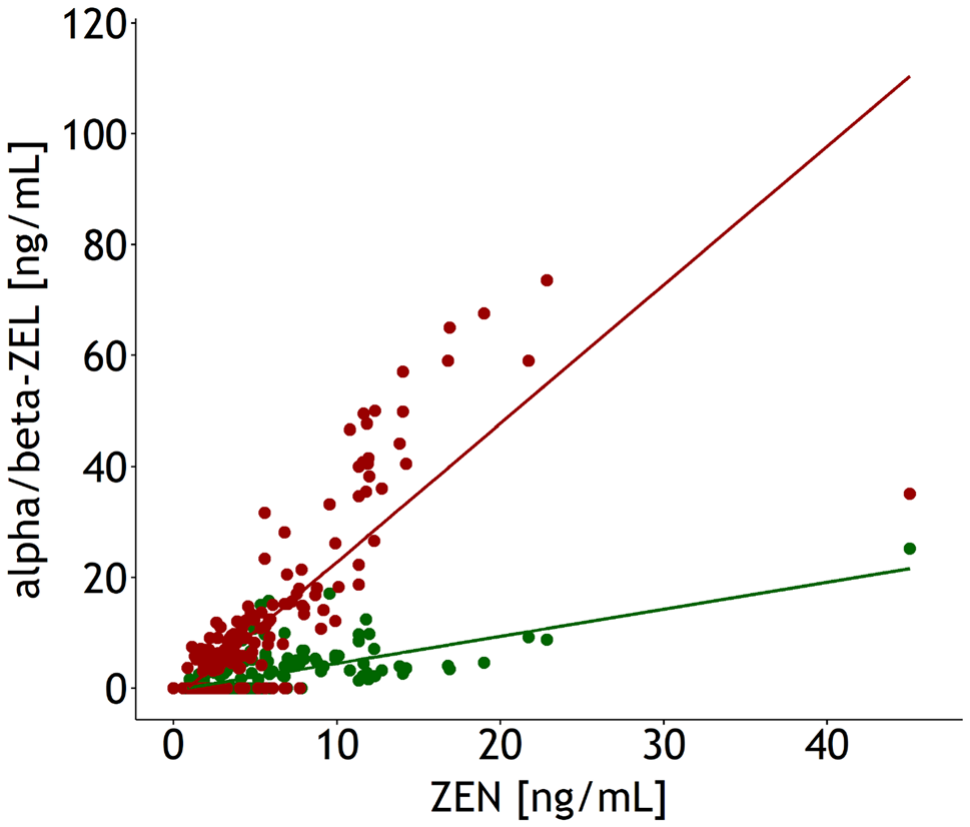
Alpha- and beta-zearalenol (ZEL) concentrations in urine in dependence on zearalenone (ZEN): alpha-ZEL = − 0.4^p>0.05^ + 0.49^p<0.05^·*x*, *r*^2^ = 0.444^p<0.05^, residual standard error = 2.55 ng/mL; beta-ZEL = − 2.22^p<0.05^ + 2.5^p<0.05^·x, *r*^2^ = 0.665 ^p<0.05^, residual standard error = 8.36 ng/mL

The slopes of the linear regressions of alpha- and beta-ZEL concentrations in urine on the ZEN concentrations suggested that alpha- and beta-ZEL concentrations increased by 0.49 and 2.5 ng/mL for each increase in urinary ZEN concentration by 1 ng/mL (Fig. 10).

## Discussion

### Farm effects on toxin exposure

The present study clearly indicated significant farm differences for the inner exposure of dairy cows to DON and ZEN when their residue levels in blood and urine were used as indicators confirming our hypothesis that feeding and feedstuffs and consequently their mycotoxin contamination levels are largely dependent on the farm. Given the fact that different geographic regions of farms implicate differences in crop production profiles and specific weather condition-dependent variations in mold and mycotoxin contamination, the results of the present mycotoxin screening of blood and urine from cows of 12 farms reflect the mycotoxin contamination status of the particular feed bases. Although the main proportion of the daily rations was based on on-farm produced feedstuffs, a smaller part of feedstuffs was bought from outside whereby mycotoxins could be “imported.”

While inner DON exposure increased with the percentage of maize silage in the ration, ZEN exposure was obviously not related to maize silage proportions. Other useful correlations between diet compositions and the inner toxin exposure were not detected supporting the view that maize and maize-based feedstuffs such as maize silage are important sources for DON which is the reason why the European Commission recommended guidance values for critical DON concentrations in maize-based feedstuffs, including maize silage, of 12 mg/kg. Although DON and ZEN frequently co-occur in maize-based feedstuffs, the dynamics of their formation in the field might be different as shown for wheat (Matthäus et al. 2004) where the increase in DON concentration was detected much earlier in relation to harvest as compared to ZEN. Assuming that such variations also apply to maize, it might be hypothesized that the phase of the pre-harvest increase in ZEN formation was not reached in the present study. This might explain why we failed to find a relevant correlation between maize silage proportion and the inner exposure to ZEN.

Although feedstuff contamination was discussed as the main source for the farm effects on the inner exposure to DON and ZEN, it should be noted that other factors might further modify their concentrations and profiles in blood and urine as mediated by the ruminal microbiota. As ruminal microbes are the key players in the pre-systemic metabolism of ZEN and DON (Dänicke and Brezina 2013; Dänicke and Winkler 2015; Gallo et al. 2022), it seems reasonable to assume that alterations in ruminal microbiota also contribute to the variation of the inner exposure to DON and ZEN. For example, age or parity and genetic background are known to alter ruminal microbial profiles (Liu et al. 2021) besides the effects of mycotoxins on ruminal microbiota itself (Boguhn et al. 2010; Hartinger et al. 2022; Strobel et al. 2008).

The weak but significant positive correlation between ZEN residues in plasma and the diet proportion of the feed fraction concentrate feed/wet maize grains/spent grains/pressed pulp was largely influenced by farm E which demonstrated distinctively higher plasma ZEN levels compared to all other farms. In this farm, the highest proportion of this feed fraction was incorporated in the TMR. Although this feed fraction cannot be characterized either in more detail or in terms of ZEN concentration, there is a high probability that ZEN contamination is traced back to pressed pulp, a byproduct of the sugar beet processing industry. Interestingly, a screening of sugar beet fibers (pulp) with a moisture content of 6% collected in Minnesota revealed ZEN as the most prevalent *Fusarium* toxin reaching concentrations up to 4.65 mg ZEN/kg DM (Bosch and Mirocha 1992). Detection of ZEN coincided with high proportions of *F. equiseti* isolates in sugar beets and fibers, an observation which could also be confirmed for sugar beets sampled in Lower Saxony after harvest and stored for different times (Christ et al. 2011). Besides, several other *Fusarium* species including *F. graminearum* and *F. culmorum* were detected. Interestingly, the latter two species produced ZEN on autoclaved rice while *F. equiseti* failed to synthesize ZEN under the tested conditions. These earlier findings became practical relevance for the European sugar beet-byproduct feedstuff market since the campaign 2018/2019 where a screening of the European Association of Sugar Manufacturers (CEFS) revealed that 41 out of 587 samples (pressed beet pulp, dried beet pulp, and pellets) contained high concentrations of 1 mg ZEN/kg or more. Since cows of the present study were sampled by the end of 2018 and the beginning of 2019 and because farm E presumably fed diets with the highest proportion of pressed sugar beet pulp, this feedstuff might have been causative for inducing the high inner ZEN exposure of cows in this farm. According to CEFS, the introduction of guidance levels for ZEN concentrations in sugar beet-based feedstuffs is currently being discussed at the European Commission (Otto 2021) making clear that ZEN contaminations of these feedstuffs might indeed pose a risk to farm and pet animals.

### Effects of lactational stage on toxin exposure

Besides the farm effect on DON and ZEN exposure, we further hypothesized that different lactational stages of cows (PP vs. EL cows) would modify the inner exposure at the same farm. Although the estimated DMI was approximately 20% lower in PP compared to EL cows, their mycotoxin residue levels in blood and urine did not differ from their corresponding farm counterparts. If it is furthermore considered that BW differences of farm corresponding PP and EL cows were small and additionally that the contamination level of feed was largely determined by farm, the similar inner exposure of PP und EL cows hints at differences in toxicokinetics. A lower DM and consequently mycotoxin intake would be associated with a longer ruminal mean retention time of ingesta and consequently more time available for ruminal mycotoxin metabolism and possibly absorption. Although we failed to demonstrate differences in blood and urine proportion of ruminally originating de-DON of the sum of DON plus de-DON between PP and EL cows due to the low positive rate of DON in both matrices, other aspects of toxicokinetics such as mycotoxin absorption efficiency or liver metabolism might be responsible. It is well known that EL cows suffer from hepatosteatosis due to the negative energy balance (NEB) (Bobe et al. 2004) with possible consequences for hepatic mycotoxin metabolism and both biliary and renal elimination. Fatty-infiltrated hepatocytes might be less efficient for phase 2-mediated conjugations whereby the plasma retention time of the unconjugated mycotoxins would be increased. However, this hypothesis needs to be proven.

### Methodological aspects of estimating the outer exposure from inner exposure

The outer exposure to DON and ZEN was expressed as their concentrations in feed to enable a comparison with the corresponding levels regarded as critical for health and performance. In doing so, two methods were applied: both using the toxin residue levels in blood or urine as the predictor variables, and outer exposure expressed either as µg toxin/kg BW/day or as dietary toxin concentration as the response variable. While the first method requires the knowledge of both BW and DMI to express exposure on a diet concentration level, the second method provides this information directly. As extensively discussed by Dänicke et al. (2023), an expression of outer exposure on a BW basis is more appropriate on toxicokinetic backgrounds but neither BW nor DMI are usually known under practical conditions and need to be estimated based on available information such as FCM, WOL, breed, and parity which might be a source of additional variation. On the other hand, the direct estimation of dietary toxin concentration neglects the possible effects of varying BW and DMI on toxin residue levels in blood and urine. Having these uncertainties of both methods in mind, the estimation of the CCC and the presentation of the corresponding Bland–Altman plots help to evaluate the differences between both methods. Surprisingly, the concordance between both methods was satisfactory as indicated by negligible deviations from the angle bisector when corresponding urine and blood estimations were considered. The Bland–Altman plots supported these conclusions although increasing dietary toxin concentrations also systematically increased the differences between both methods suggesting that variation in estimation of dietary DON and ZEN concentration increases with corresponding dietary levels irrespective of the method used as supported by the symmetric distribution of observations in the Bland–Altman plots. Based on these methods’ comparison, it can be concluded that the direct estimation of DON and ZEN levels of diets without the detour of additional estimation of BW and DMI might match practical usefulness without compromising accuracy.

### Risk assessment based on estimated outer toxin exposure

Taking the findings for the estimated diet concentrations of DON and ZEN collectively, we noticed significant variations amongst the farms at levels lower than the critical diet concentration of 5 mg DON/kg and small variations in ZEN concentrations except for one farm exceeding the guidance value of 0.5 ZEN mg/kg.

Although predicted dietary DON levels varied well below the guidance value of 5 mg/kg diet, the toxicological relevance might be questioned in the view of recent findings describing a role of de-DON in biochemical effects different from the mode of action of DON. This includes effects on steroid hormone metabolism and induction of endoplasmic reticulum stress in primary bovine theca cells (Guerrero-Netro et al. 2017; Reyes-Perea et al. 2023). Comparably low concentrations of 0.5 ng DON or de-DON/mL in the medium of cultured bovine theca cells inhibited progesterone synthesis while only de-DON induced additional apoptosis at a level of 1 ng/mL. Moreover, unlike DON, de-DON enhanced the mRNA expression of the endoplasmic reticulum (ER) stress-associated proteins PRKRA and ATF4 (Guerrero-Netro et al. 2017). Translating these medium de-DON and DON concentrations into the real-life situation, such toxin levels are expectable under practical feeding conditions in bovine follicular fluid (Winkler et al. 2014a).

Based on a proteomics approach, Torabi et al. (2021) concluded from their experiments with in vitro cultivated theca cells that low levels of 1 ng/mL DON or de-DON can activate mitogen-induced proliferative molecules capable of stimulating tumorigenesis in the ovary. Based on the analyses of follicular fluid from cows exposed to graded levels of DON in the diet (Winkler et al. 2014a), the concentrations used in the mentioned in vitro experiment would correspond to dietary DON levels of approximately 2 to 5 mg/kg. One micromole of each DON and ZEN (equivalent to 296 and 323 ng/mL) induced an apoptotic phenotype of in vitro cultured bovine theca cells while de-DON failed to be effective at this concentration (Cai et al. 2023). Moreover, levels of 0.01 and 1 µM of DON and de-DON (equivalent to 3 and 280 ng/mL) induced NLRP3 inflammasome and related genes. These effects were even potentiated when theca cells were co-exposed with DON and de-DON at the mentioned concentrations of 0.01 and 1 µM, respectively (Cai et al. 2023). Referring again to the study by Winkler et al. (2014a), such concentrations might occur in follicular fluid under in vivo conditions.

Taking the discussed in vitro effects of de-DON on bovine theca cells together and given the fact that de-DON nearly equilibrates with plasma de-DON, it can be concluded from the present study that effective in vitro de-DON levels of 0.5–1 ng/mL were exceeded in plasma, and consequently presumably also in follicular fluid, of most cows sampled in the present study. The relevance of this situation for the in vivo fertility of cows needs to be examined in more detail as only the NRR as a reproductive parameter was recorded in the present study. Based on the limited number of cows available for an evaluation of the NRR within the scope of the present study, the insignificant farm and group effects together with the significant farm differences in the inner exposure to de-DON (blood), and its weak and insignificant correlation to the NRR (*r* = − 0.03, *p* > 0.05), no valid conclusion can be drawn on possible effects of de-DON on cow’s fertility. It has additionally to be taken into account that the reproductive performance of cows is multi-factorially influenced. Moreover, the cows left the farms for infertility showed de-DON concentrations in urine comparable to those kept on farms.

In contrast to de-DON and DON, the role of ZEN as an endocrine disruptor interfering with fertility particularly of female animals is well known (European Food Safety Authority 2017) although cows are regarded as less sensitive because of the effective conversion of ZEN to the less estrogenically active beta-ZEL (European Food Safety Authority 2017). This efficient conversion could also be confirmed by the present study as indicated by the 2.5-fold increase in urinary beta-ZEL compared to ZEN. However, recent screening of urine and feed from Canadian farms suggested both ZEN and beta-ZEL to be associated with reproductive and health traits for dairy cows (Tazerout 2016). For example, ZEN concentrations in urine higher than 2.7 ng/mL were associated with a significantly increased timespan until the next pregnancy. In the present study, 7 out of 12 farms imposed median urinary ZEN concentrations higher than 2.7 ng/mL (data not shown). However, based on the limited observations of reproductive traits, no valid conclusion on the relevance of this situation can be drawn. Besides apparent associations between ZEN concentration in urine and calving interval, a beta-ZEL content of higher than 6.23 ng/mL in urine was related to a significantly higher proportion of cows leaving the farms irrespective of the cause. The threshold of 6.23 ng beta-ZEL/mL urine was exceeded by 6 out of the 12 farms when the median was considered for evaluation. However, the individual distribution of beta-ZEL concentrations of cows left the farms compared to those kept on the farms was not different.

Mammary alveolar cells exposed in vitro to DON concentrations of 1 µM (= 296 ng/mL) and more responded with a decreased proliferation, β-casein, and lipid droplet synthesis, effects that were related to a disruption in tight junction proteins (Zhao et al. 2022). It needs to be stressed that even the lowest tested DON concentration in this in vitro study was manifold higher than those levels recorded in milk (up to 2.5 ng/mL) collected from feeding trials covering dietary DON levels up to approximately the guidance value of 5 mg DON/kg (Keese et al. 2008; Seeling et al. 2006; Winkler et al. 2015a). Neither FCM nor milk fat and protein contents were correlated to any of the DON residue-derived parameters of the present study suggesting that the significant variation of the estimated DON contents was without relevance for in vivo inhibition of synthesis of milk and milk components.

## Limitations of the study

In overall discussing the results of the present study, it needs to be considered that cows were sampled only once for the purpose of evaluation of DON and ZEN exposure. Although feedstuff batches used for preparing the TMR are usually fed over weeks or even months, the DON and ZEN concentrations might be subject to changes over time particularly due to inhomogeneous toxin distributions within batches. Therefore, the here estimated DON and ZEN concentrations in the TMR reflect the actual feed contamination level and can indicate the longer-term situation only with an unknown degree of uncertainty. Therefore, the evaluated associations to production traits of cows which were recorded around the time point of blood and urine sampling might also bear uncertainty.

## Conclusions

Considering the discussed limitations of the study, there were no hints at significant associations of significant farm-related differences in inner and outer exposure to DON and ZEN to production, reproduction, and health traits of cows. Within farms, there were no differences in mycotoxin exposure between PP and EL cows which was discussed to reflect the interplay between physiological mechanisms regulating feed intake including ruminal ingesta and toxin retention time with consequences for pre-systemic toxin metabolism.

For a more robust evaluation of possible relationships between DON/ZEN exposure and production, reproduction, and health traits of cows, longitudinal screenings are necessary with repeated sampling of cows and feed for mycotoxin concentrations and parallel recording of animal traits. Such a screening appears to be particularly important to evaluate the in vivo relevance of reported in vitro results suggesting toxic effects both of DON and de-DON on bovine theca cells at medium concentrations also observed in plasma samples of the present study.

## Data Availability

No datasets were generated or analyzed during the current study.
